# A flax fibre proteome: identification of proteins enriched in bast fibres

**DOI:** 10.1186/1471-2229-8-52

**Published:** 2008-04-30

**Authors:** Naomi SC Hotte, Michael K Deyholos

**Affiliations:** 1 Department of Biological Sciences, Edmonton, T6G 2E9, Canada

## Abstract

**Background:**

Bast fibres from the phloem tissues of flax are scientifically interesting and economically useful due in part to a dynamic system of secondary cell wall deposition. To better understand the molecular mechanisms underlying the process of cell wall development in flax, we extracted proteins from individually dissected phloem fibres (i.e. individual cells) at an early stage of secondary cell wall development, and compared these extracts to protein extracts from surrounding, non-fibre cells of the cortex, using fluorescent (DiGE) labels and 2D-gel electrophoresis, with identities assigned to some proteins by mass spectrometry.

**Results:**

The abundance of many proteins in fibres was notably different from the surrounding non-fibre cells of the cortex, with approximately 13% of the 1,850 detectable spots being significantly (> 1.5 fold, p ≤ 0.05) enriched in fibres. Following mass spectrometry, we assigned identity to 114 spots, of which 51 were significantly enriched in fibres. We observed that a K^+ ^channel subunit, annexins, porins, secretory pathway components, β-amylase, β-galactosidase and pectin and galactan biosynthetic enzymes were among the most highly enriched proteins detected in developing flax fibres, with many of these proteins showing electrophoretic patterns consistent with post-translational modifications.

**Conclusion:**

The fibre-enriched proteins we identified are consistent with the dynamic process of secondary wall deposition previously suggested by histological and biochemical analyses, and particularly the importance of galactans and the secretory pathway in this process. The apparent abundance of β-amylase suggests that starch may be an unappreciated source of materials for cell wall biogenesis in flax bast fibres. Furthermore, our observations confirm previous reports that correlate accumulation proteins such as annexins, and specific heat shock proteins with secondary cell wall deposition.

## Background

Flax (*Linum usitatissimum *L.) has attracted human attention since the beginning of agriculture [1,2]. This is due in part to the unusual properties of the bast (i.e. phloem) fibres, which because of their great length and high tensile strength have found use in textiles and many other products [3]. Fibre length is achieved almost entirely through intrusive growth, which is a process limited to very few cell types in plants [4,5]. The elongation stage is succeeded by a dynamic process of secondary wall deposition, in which a matrix of galactose-rich polymer in the nascent wall is gradually and centripetally replaced by highly crystalline cellulose [6]. Because secondary wall deposition increases the tensile strength of cells, fibres which have undergone even the very first stages of cell wall thickening can be distinguished mechanically by their resistance to breakage at the "snap-point" of the stem [7]. The snap-point thus defines an important developmental transition from cell elongation to cell wall thickening.

Previously, we and others have produced libraries of cDNAs from fibre-bearing peels of flax and hemp stems [8,9]. In addition to containing bast fibres at various stages of development, these peels also contained many other cell types, including those associated with cambium and transport phloem. Analysis of these libraries by cDNA microarray hybridization and other techniques identified distinct patterns of expression of transcripts of polysaccharide-related enzymes in stem peels during fibre elongation and cell wall deposition. However, due to inherent technical and biological limitations, it is known that in many circumstances, abundance of transcripts and proteins for a given gene may not be highly correlated [10,11]. This well-established limitation on the biological relevance of transcriptome analysis led us to complement our previous work with a survey of the proteins present in developing flax fibres during the onset of secondary wall deposition. This is similar to a proteomics approaches used to study secondary cell wall development of other cell types in other species [12-16]. For this study of the proteome, we also increased the specificity of our analysis by extracting proteins from phloem fibres that had been individually dissected from the snap point of growing stems, and comparing their abundance to proteins in the surrounding, non-fibre cells of the cortex from the same stems. The objective of this study is therefore to identify those proteins that contribute to the interesting pattern of cell wall deposition in flax fibres.

## Results and discussion

### Separation of fibre and non-fibre proteins

**Figure 1:**
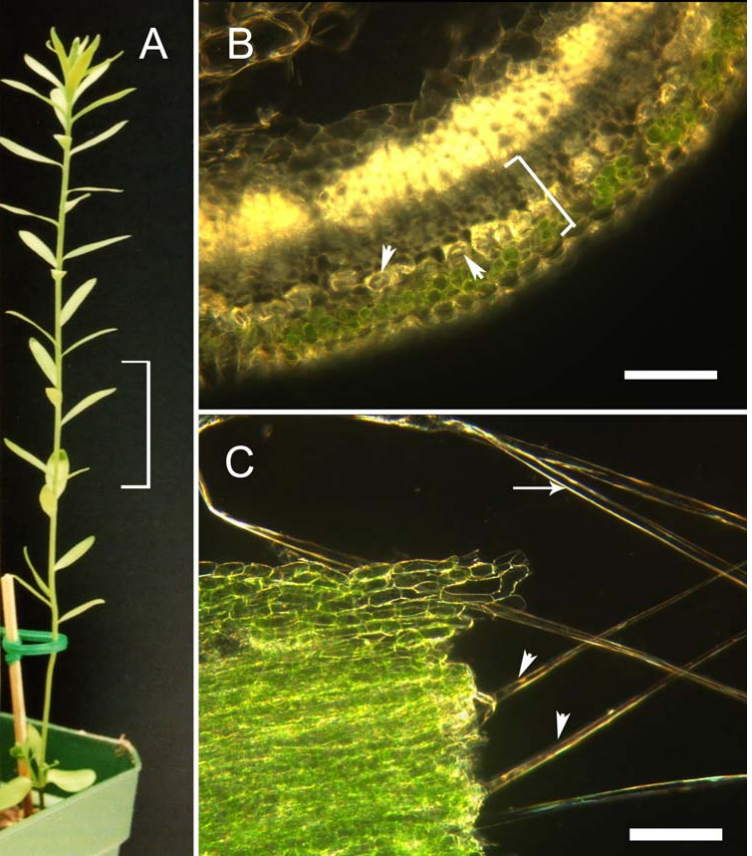
**A typical flax plant at the time of fibre extraction**. (A) The 3 cm region of the stem from which fibres were dissected is indicated by the bracket. (B) Detail of a transverse section of fresh stem tissues at the time of harvest. This hand section was obtained from just below the snap-point to demonstrate the arrangement of tissues within the stem, i.e. transverse sectioning was not used when obtaining tissues for protein analysis. A bracket indicates the region of the cortex from which the fibre and non-fibre fractions would be obtained. The position of representative fibres within the cortex is shown by arrowheads. The scale bar is 100 μm. (C) Stem tissues during dissection. Fibres from which surrounding, non-fibres cells been partially removed are indicated by arrowheads. A fully dissected fibre, comprising a single cell is indicated by the arrow. This fibre is representative of the cells from which proteins were extracted. The scale bar is 100 μm.

**Figure 2:**
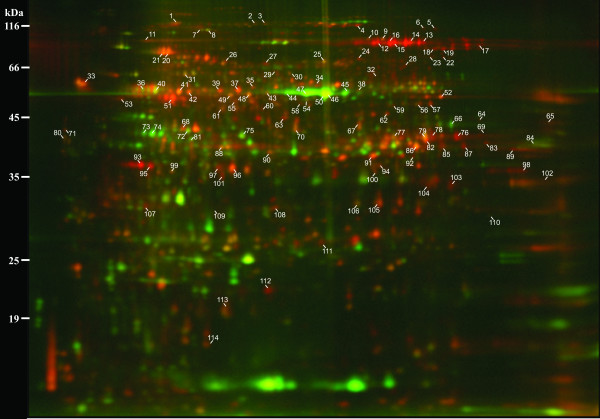
**Representative analytical DiGE gel**. Proteins extracted from fibre and surrounding non-fibre tissues were fluorescently labeled with red and green dyes, respectively, and were mixed then separated simultaneously using 2D gel electrophoresis. Labels correspond to protein spot numbers used in Table 1 and in the text. The pH range of the first dimension separation is from 3 (left) to 10 (right).

To increase our understanding of the proteins that contribute to the unique properties of flax bast fibres, we extracted proteins from ultimate fibres (i.e. individual cells) dissected from the snap-point region of vegetative stems (21–24 days post germination) (Figure 1). The snap-point is the stem region in which secondary wall deposition begins [7]. We also collected the surrounding non-fibre cells (consisting predominantly of parenchyma, sieve elements, and companion cells) from the cortex of the snap-point. Throughout the remainder of this report, will refer to the ultimate bast fibres we collected from the snap-point as simply "fibres", and the surrounding, non-fibre cells of the cortex as the "non-fibre fraction". By labelling proteins from fibres and the non-fibre fraction with contrasting fluorescent dyes, and separating the mixture of the two samples simultaneously using 2D gel electrophoresis (DiGE), we were able to identify proteins that were more abundant in fibres as compared to the non-fibre fraction (Figure 2).

**Figure 3:**
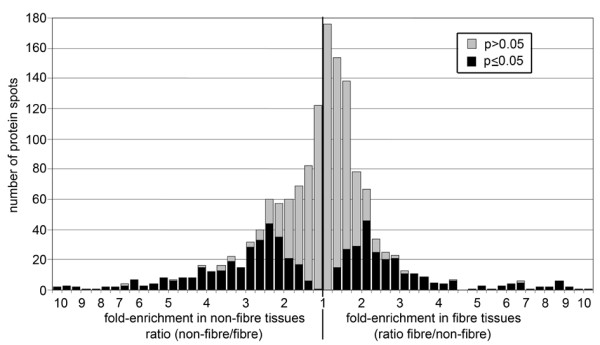
**Frequency distribution of mean intensity ratios for all spots**. A mean ratio near 1 meant the spot was found in equal abundance in both tissues; spots represented to the right of this point on the axis had higher signal intensity in fibre tissues, while spots represented to the left were more intense in non-fibre tissues. The grey and black regions of each bar show the portion of spots for which p > 0.05 and p ≤ 0.05, respectively, in a t-test of the significance of differences in intensity between fibre and non-fibre tissues.

In each of four replicate gels, we detected an average of 1850 distinct protein spots from fibres, and 1695 spots from the non-fibre fraction. In total, 558 protein spots differed in fluorescent signal intensity by at least 1.5 fold (p ≤ 0.05) between the samples, with 246 spots (13% of total detected) enriched in fibres and 312 spots (18% of total) enriched in the non-fibre fraction (Figure 3). The distinctive protein profiles of fibres and the non-fibre fraction were also evident from visual inspection of the DiGE gel image (Figure 2). Phloem fibres therefore appear to express a complement of proteins that is distinct from surrounding cell types in the stem.

### Protein identification by LC/MS

We picked 190 protein spots that were enriched in fibre samples for identification by mass spectrometry. Spots were selected based on criteria of large spot volume, high fold-enrichment of signals, and well-focused spot morphology. For comparison, we also selected an additional 50 spots that were enriched in non-fibre fractions or that were similarly abundant in both types of protein samples. Although the patterns of fold-enrichment that we report were reproducible within the statistical parameters indicated (Table 1), individual ratios should not be extrapolated quantitatively to whole proteins, in part because some proteins may be represented by more than one spot.

**Table 1:** Protein identities based on peptide matches to Genbank protein databases

				fold enrich.^b^				
spot ID#	func. cat.^a^	protein identity	Genbank ID	fibre	non-fibre	p-value^c^	Mowse score	pep. count^d^

2	C&E	aconitate hydratase	4586021	1.5		0.14	64	2
3	C&E	aconitate hydratase	4586021	1.5		0.08	68	2
17	C&E	β-amylase	1771782	8.8		< 0.01	46	2
39	C&E	ribulose-1,5-bisphosphate carboxylase/oxygenase large subunit	168312	1.5		0.25	85	2
40	C&E	ribulose-1,5-bisphosphate carboxylase, large subunit	168312		2.0^e^	0.08	180	4
44	C&E	ribulose-1,5-bisphosphate carboxylase, large subunit	1834444		6.1	< 0.01	129	5
45	C&E	ribulose-1,5-bisphosphate carboxylase, large subunit	2687483		5.4	< 0.01	130	4
46	C&E	ribulose-1,5-bisphosphate carboxylase, large subunit	6983900		2.9	< 0.01	232	6
47	C&E	ribulose-1,5-bisphosphate carboxylase, large subunit	1817560		3.3	< 0.01	250	5
48	C&E	enolase	9581744	1.1		0.65	265	7
49	C&E	enolase	8919731		1.1	0.93	158	3
50	C&E	enolase	9581744		3.4	0.02	206	6
51	C&E	ribulose-1,5-bisphosphate carboxylase, large subunit	4098530	2.8		0.04	103	4
57	C&E	fumarate hydratase	108708038	2.5		0.01	83	2
58	C&E	fumarate hydratase	15226618	1.6		0.33	100	4
59	C&E	6-phosphogluconate dehydrogenase	2529229	1.5		0.19	100	3
63	C&E	citrate synthase	11066954	3.7		< 0.01	123	4
68	C&E	phosphoglycerate kinase	1161600	1.2		0.56	257	4
71	C&E	phosphoglycerate kinase	92872324	1.7		0.06	426	7
72	C&E	ribulose-1,5-bisphosphate carboxylase/oxygenase large subunit	66735801				96	3
73	C&E	rubisco activase	13430332		6.1	< 0.01	70	3
74	C&E	rubisco activase	170129		5.2	< 0.01	61	3
75	C&E	phosphoglycerate kinase	3328122		2.9	0.02	250	6
77	C&E	fructose-bisphosphate aldolase	15227981	1.1		0.82	155	3
78	C&E	fructose-bisphosphate aldolase	20204	2.4		0.03	102	2
79	C&E	fructose-bisphosphate aldolase	15227981	1.1		0.6	116	2
80	C&E	fructose-bisphosphate aldolase	20204	1.3		0.04	177	3
81	C&E	rubisco activase	4261547		2.2	0.03	60	2
82	C&E	succinate-CoA ligase	15225353	2.3		0.02	253	5
83	C&E	glyceraldehyde-3-phosphate dehydrogenase	120666	2.6		0.01	76	2
86	C&E	glyceraldehyde-3-phosphate dehydrogenase	3023813	1.1		0.49	71	3
87	C&E	glyceraldehyde-3-phosphate dehydrogenase	74419004	3.8		< 0.01	215	6
90	C&E	malate dehydrogenase	18297	1.6		0.17	241	4
91	C&E	malate dehydrogenase	18297	1.4		0.26	138	4
92	C&E	malate dehydrogenase	10334493	3.3		< 0.01	296	7
93	C&E	fructokinase	31652274	6.7		< 0.01	142	5
94	C&E	fructokinase	31652274	2.2		< 0.01	154	3
96	C&E	kinase/ribokinase, potential fructokinase	15224669	2		0.01	208	8
1	ATP	AAA-ATPase	86212372	1.6		0.24	322	10
7	ATP	ATPase, transitional endoplasmic reticulum	7378614	1.2^e^		0.65	101	4
24	ATP	vacuolar proton-ATPase	50251203	2.6		0.02	585	13
31	ATP	ATP binding	15221770		1	0.87	100	4
35	ATP	F1 ATPase	12986	1.6		0.05	143	6
40	ATP	ATP synthase β subunit	21684923		2.0^e^	0.08	192	4
42	ATP	ATP synthase β subunit	19685	1		0.99	675	12
43	ATP	ATP synthase β subunit	56784991		1.8	0.06	307	7
99	ATP	F1-ATPase gammma subunit	303626	1		0.66	84	3
105	ATP	vacuolar V-H^+^ATPase subunit E	5733660	1.8		0.01	53	2
106	ATP	vacuolar V-H^+^ATPase subunit E	5733660		1.1	0.82	100	4
12	CWP	β-galactosidase	115437888	8.4		< 0.01	43	3
13	CWP	β-galactosidase	3641863	8.9		< 0.01	42	2
14	CWP	β-galactosidase	3641863	5.4		< 0.01	105	5
15	CWP	β-galactosidase	3641863	8.8		< 0.01	96	5
16	CWP	β-galactosidase	34913072	9.3		< 0.01	72	4
18	CWP	*MUCILAGE-MODIFIED 4*	42562732	4.1		< 0.01	57	2
19	CWP	rhamnose biosynthetic enzyme	108707484	6.6		< 0.01	100	6
27	CWP	phosphoglucomutase	12585309	1.8		0.15	170	4
28	CWP	phosphoglucomutase	6272281	3.7		0.02	122	5
36	CWP	UDP-glucose pyrophosphorylase	6136112		1.4	0.37	82	3
38	CWP	UDP-glucose pyrophosphorylase	82659609		3.5	0.01	166	6
41	CWP	UDP-glucose pyrophosphorylase	9280626	1.6^e^		0.1	129	6
64	CWP	β-galactosidase	3641863	1.2		0.51	72	2
76	CWP	NAD-dependent epimerase/dehydratase (UXS6)	15226950	6.1		< 0.01	109	4
88	CWP	UDP-glucose 4-epimerase	12643850		1.1	0.84	60	2
101	CWP	GDP-4-keto-6-deoxy-D-mannose-3,5-epimerase-4-reductase	12324315	2.3		< 0.01	155	3
104	CWP	dTDP-D-glucose 4,6-dehydratase-like	50253123	3		< 0.01	56	2
9	1C	Met synthase	77556633	2		< 0.01	222	6
10	1C	Met synthase	8439545	2.2		< 0.01	105	3
41	1C	S-adenosyl-L-homocysteine hydrolase	1710838	1.6^e^		0.1	174	5
53	1C	serine hydroxymethyltransferase	11762130	2.2		0.02	129	4
60	1C	Met adenosyltransferase	37051117	2.1		0.02	94	4
55	MemT	GDP dissociation inhibitor	8439465	2		0.13	212	5
56	MemT	GDP dissociation inhibitor	8439465	1.9		0.08	158	4
95	MemT	K^+ ^channel β-subunit	15219795	8.6		0.01	132	4
102	MemT	34 kDa outer mitochondrial membrane porin-like protein	83283993	1.7			55	2
103	MemT	36kDa porin I	515358	3.9		< 0.01	104	4
5	C&S	myosin heavy chain	108710464	2.5		0.05	46	2
6	C&S	myosin heavy chain	T00727	3.6		0.01	48	2
22	C&S	dynamin central region	92891191	3.1		0.09	83	3
25	C&S	dynamin-like	21593776		1	0.77	143	4
37	C&S	β-tubulin	295851	1.8		0.06	161	6
52	C&S	tubulin/FtsZ family, GTPase domain	62734655	1.7		0.06	367	12
69	C&S	actin	32186910	3.1		0.01	281	8
70	C&S	actin	15242516	1.5		0.21	459	12
4	P&AA	elongation factor EF-2	6056373	2.5		0.02	40	3
7	P&AA	ClpC protease	4105131	1.2^e^			81	4
8	P&AA	ClpC protease	18423214		1.7	0.06	286	11
11	P&AA	HSP 90	1708314	1.7		0.01	312	10
20	P&AA	HSP 70-3	38325815	1.9		0.08	404	11
21	P&AA	HSP 70	62733235	1.7		0.11	612	13
23	P&AA	HSP 70	22636	2		0.04	100	3
29	P&AA	chaperonin CPN60-1	108706134	2.7		0.04	139	6
30	P&AA	chaperonin CPN60-1	108706134	1.5		0.04	327	7
32	P&AA	HSP 60	16221	2.1		0.02	140	4
54	P&AA	eukaryotic elongation factor 1A	24371059		2.3	0.02	227	7
61	P&AA	26S protease regulatory subunit	1709798	2.1		< 0.01	85	3
62	P&AA	translation initiation factor eIF-4A	475221	1.6		0.09	262	9
65	P&AA	26S proteasome subunit P45	92870338	1.9		0.11	90	3
66	P&AA	aminomethyltransferase	3334196		3.7	< 0.01	67	2
67	P&AA	elongation factor-1 alpha	396134	1.2		0.5	54	3
72	P&AA	glutamine synthetase	121341		1.7^e^	0.26	119	4
84	P&AA	P0 ribosomal protein	1143507		2.5	< 0.01	155	3
89	P&AA	glutamate-ammonia ligase	99698	1.2^e^		0.5	65	3
114	P&AA	eukaryotic translation initiation factor 5A	8778393	2		0.04	91	2
26	misc	nucleolar protein NOP5	108708132	1.4		0.37	47	2
33	misc	ferric leghemoglobin reductase	5823556	1.6		0.2	124	4
34	misc	calreticulin	3288109		1	0.9	78	3
85	misc	peroxidase	1389835	2.4		0.03	214	7
89	misc	type IIIa membrane protein cp-wap13	2218152				58	3
97	misc	annexin	1429207	2.2		0.01	146	4
98	misc	annexin	1429207	4.1		0.03	71	2
100	misc	enoyl-ACP reductase	2204236	2.1		0.01	44	2
107	misc	protein kinase C inhibitor	20062	2.8		< 0.01	97	5
108	misc	14-3-3 protein	695767	2.7		0.01	44	3
109	misc	guanine nucleotide regulatory protein	395072	1.5		0.31	64	2
110	misc	NAD(P)H dependent 6'-deoxychalcone synthase	18728		1.1	0.82	56	3
111	misc	inorganic pyrophosphatase	48927683	2.8		0.02	148	3
112	misc	maturase K	33332553	3.4		0.01	55	2
113	misc	CBS (cystathionine β-synthase) domain-containing	15238284	1.6		0.1	92	2

a) Functional category: ATPases (ATP); Cell wall polysaccharide metabolism (CWP); Cytoskeleton and secretion (C&S); Membrane transport (MemT); Miscellaneous (misc); One-carbon metabolism (1C); Primary carbon and energy metabolism (C&E); Protein and amino acid metabolism (P&AA). Only the highest scoring protein for each spot is categorized.

b) Fold enrichment in fibre tissues or non-fibre tissues as compared to the other tissue type, expressed as linear ratio of mean signal intensities.

c) P-value for a t-test of significant differences in mean signal intensities between fibre and non-fibre tissues.

d) Peptide count, i.e. the number of peptides per spot that match the Genbank ID shown.

e) Spots in which multiple proteins were identified. The intensity ratios shown may be due to differences in abundance of more than one protein.

Protein identities are sorted by functional category, in the order in which each category is presented in the text, and then alphabetically within each functional category. Additional data (including peptide sequences) is provided in Additional File 1.

We subjected a total of 240 spots to analysis by LC/MS. Of these, 126 spots produced spectra that could not be assigned to existing sequences, while spectra from the remaining 114 spots produced significant matches (i.e. MOWSE scores 40–675; two or more peptides matched per spot) to predicted spectra from Genbank protein databases (Table 1). Four spots (#7, #41, #72, #89) contained predicted peptides that matched more than one distinct protein, indicating the presence of multiple proteins in some spots on the gel. Of the spots to which we assigned protein identities, 76 were enriched by at least 1.5 fold (i.e. 1.5×) in fibre samples, and 51 of these were statistically more abundant (p ≤ 0.05) in fibres than the non-fibre fraction. Conversely, we were able to assign identity to 17 spots enriched 1.5-fold or more in the non-fibre fraction; at least seven of these were associated with photosynthesis (spots #44–#47, #73, #74, #81). Because photosynthesis is a process expected to dominate metabolism in the non-fibre fraction, these observations are consistent with the physical separation of fibre and non-fibre tissues we hoped to achieve by dissection. We will focus the remainder of this report on the spots that were enriched in fibres.

**Figure 4:**
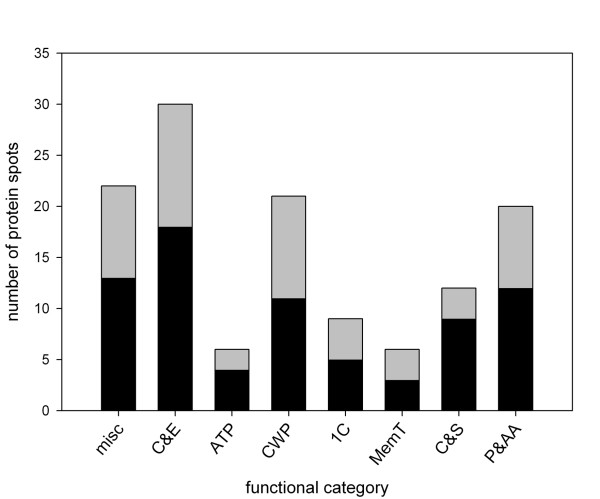
**Functional categorization of fibre-enriched proteins**. All spots for which signal intensity was at least 1.5-fold greater in fibres as compared to non-fibres, and for which identity could be assigned by MS, were assigned to one of the categories shown. The grey and black regions of each bar show the portion of spots for which p > 0.05 and p ≤ 0.05, respectively, in a t-test of the significance of differences in intensity between fibre and non-fibre tissues. ATPases (ATP); Cell wall polysaccharide metabolism (CWP); Cytoskeleton and secretion (C&S); Membrane transport (MemT); Miscellaneous (misc); One-carbon metabolism (1C); Primary carbon and energy metabolism (C&E); Protein and amino acid metabolism (P&AA).

The fibre-enriched proteins to which we were able to assign putative identities were classified into eight functional categories (Figure 4). Aside from the category we called "miscellaneous", which represented a diverse set of functions, most of the proteins that were identified in fibre samples could be assigned to one of three categories related to the conversion of carbohydrates for energy or glycan biosynthesis, namely: primary carbon and energy metabolism; one-carbon metabolism; and cell wall and polysaccharide metabolism (Figure 4). The predominance of these proteins for the metabolism of carbohydrates and related compounds is consistent with the major biochemical activities we expected to observe within cells active in secondary wall biogenesis. In addition, we assigned a smaller number of proteins to each of the remaining categories: membrane transport; cytoskeleton & secretion; ATPases; and protein & amino acid metabolism. The membership of proteins assigned to spots in each of the eight functional categories is shown in Table 1, and is discussed in more detail in the following sections.

### Primary carbon and energy metabolism

**Figure 5:**
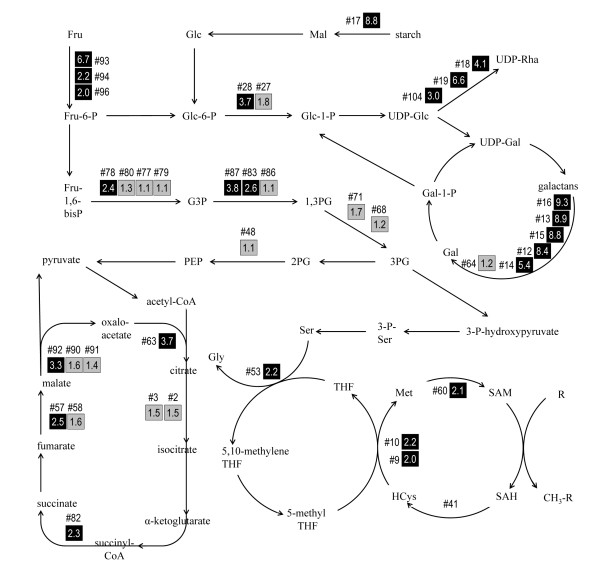
**Relative abundance of fibre-enriched proteins identified as enzymes in selected reactions of carbohydrate and one-carbon metabolism**. Numbers following the symbol '#' are the unique spot identifiers used in Table 1 and throughout the text. Values in boxes show the fold-enrichment (i.e. signal intensity in fibres/non-fibres). Grey and black filled boxes indicate spots for which p > 0.05 and p ≤ 0.05, respectively, in a t-test of the significance of differences in intensity between fibre and non-fibre tissues. No intensity ratio is shown for #41, because multiple proteins were identified within this spot. Pathways shown are based on data from KEGG and AraCyc [37, 38]. Not all reactants or co-factors are shown. Abbreviations used in names of substrates include fructose (Fru), galactose (Gal), glucose (Glc), glyceraldehyde-3-phosphate (G3P), homocysteine (HCys), maltose (Mal), phosphoglycerate (PG), phosphoenolpyruvate (PEP), rhamnose (Rha), S-adenosyl homocysteine (SAH), tetrahydrofolate (THF).

The conversion of monosaccharides and starch into energy is the inferred function of the largest proportion of proteins that were enriched (> 1.5 fold) in fibres, as compared to the non-fibre fraction at the stem snap point (Figure 4). These reactions are also summarized in Figure 5. Two of the most highly enriched proteins we detected in any functional category were β-amylase (spot #17; 8.8× fold enriched in fibres), and fructose kinase (#93, 6.7×; #94, 2.2×; #96, 2.0×), which catalyze the first steps in the catabolism of starch and fructose, respectively (Table 1). The increased relative abundance of these enzymes in fibres provides some insight into the immediate sources of carbon and energy for secondary wall biogenesis. We also detected the statistically significant (p ≤ 0.05) enrichment of enzymes of glycolysis and related processes, namely fructose-bisphosphate aldolase (#78, 2.4×), glyceraldehyde 3-phosphate dehydrogenase (#83, 2.6×; #87, 2.8×), and phosphoglucomutase (#27, 1.8×; #28, 3.7×), as well as the presence of phosphoglycerate kinase (#68, #71). Finally, we identified fibre-enriched protein spots putatively representing 5 of 8 enzymes of the tricarboxylic acid cycle, where further energy and metabolic precursors are generated from the products of glycolysis. The tricarboxylic acid cycle -associated proteins that were significantly enriched in fibres and included citrate synthase (#63, 3.7×), succinyl coA-ligase (#82, 2.3×), fumarase (#57, 2.5×), and malate dehydrogenase (#92, 3.3×). Aconitate hydratase (#2, #3) was also detected, although its fold-enrichment was not statistically significant (p > 0.05).

### ATPases

Many subunits of the ATPase/synthase complex were identified in either fibres or the non-fibre fraction, including an α-subunit (#35), β-subunits (#42, #43), and a γ-subunit (#99). The tissue-specific abundance patterns of these various subunits were surprisingly complex: the γ-subunit and one β-subunit (#42) were associated with equal spot intensities in both sample types, while the other ATP synthase β-subunit (#44), was 1.8× more abundant in the non-fibre fraction. Only the α-subunit was more abundant (1.6×) in fibres.

In addition to the ATPase/synthases described above, we identified peptides from several other types of putative ATPases, including three protein spots containing vacuolar-type ATPase (v-ATPase), of which, two spots (#24, 2.6×; #105, 1.8×) were significantly (p ≤ 0.05) enriched in fibres. v-ATPases are some of the most abundant membrane proteins in the vacuole and endomembrane system, and their enrichment may reflect increased relative abundance of these organellar structures in fibres [17]. We also detected a putative plasma membrane-associated AAA-ATPase (#1, 1.6×) in fibres, although this was not deemed to be more abundant in fibres by our usual statistical criteria. Both v-ATPases and AAA-ATPases have been previously demonstrated to be essential for vesicle transport, and might therefore be active in secondary wall-specific processes in developing fibres [17,18].

### Cell wall and polysaccharide metabolism

Cell walls consist of many types of polymers, including cellulose, hemicellulose, and pectins. However, with the possible exception of an NAD-dependent epimerase/dehydratase with similarity to UDP-xylose synthases (#76, 6.1×), and GDP-4-keto-6-deoxy-D-mannose-3,5-epimerase-4-reductase (GME, #101, 2.3×) almost all of the fibre-enriched, cell wall-related enzymes we identified were most likely associated with the metabolism of pectin-like substances. For example, we identified proteins from six spots as β-galactosidases. Five of these (#12–#16) were co-located in a charge train and the sixth (#64) was an isolated spot of lower apparent molecular weight. The five spots in the charge train were significantly more intense in fibres (5.4–9.3×), while the lower molecular weight spot was nearly similar in abundance in both types of tissues (1.15×). Within the charge train, peptides from three spots aligned with a chickpea β-galactosidase as the highest scoring match. This chickpea β-galactosidase has previously demonstrated exo- and endo- cleavage activity towards the side-chains of pectins and is found in elongating hypocotyls [19,20]. In developing flax fibres, the deposition of a rhamnogalactan-type pectin consisting of 55–85% galactose is known to precede establishment of the crystalline, cellulosic fibrils that characterize the mature secondary wall [6]. Because the galactose residues of rhamnogalactans are one of the putative substrates for β-galactosidase, we speculate that the abundance of this enzyme in developing fibres is evidence of an important role for it in remodeling, removing, or recycling of galactans as part a dynamic process of cell wall deposition. However, it is also possible that the β-galactosidase we detected hydrolyzes other galactosyl bonds, such as those that decorate arabinogalactan proteins [21]. Finally, the appearance of the β-galactosidase spots in a train along the axis of the first dimension separation of our electrophoretic gels is consistent with extensive post-translational modification of this abundant protein.

In addition to β-galactosidase, we also identified other spots representing one or more enzymes with possible roles in the metabolism of pectic polysaccharides. Three spots (#18, 4.1×; #19, 6.6×; #104, 3.0×) were more enriched in fibres as compared to the non-fibre fraction and share homology with UDP-rhamnose synthase. Because these enzymes would normally be expected to contribute to the growth of rhamnogalactans, it is interesting to observe their enrichment in the same cells in which β-galactosidase might hydrolyze galactosidic bonds within these polymers. The potential co-existence of both catabolic and anabolic processes of galactan metabolism is consistent with a rapid turnover of these polymers during cell wall deposition, although the existence of the inferred enzymatic activities must still be confirmed experimentally.

### One-carbon metabolism

Four enzymes associated with one-carbon (1C) metabolism were identified among the fibre-enriched protein spots in our study. Three of these: methionine synthase (#9, #10; 2.0×, 2.2× respectively), methionine adenosyltransferase (#60; 2.1×), and adenosylhomocysteinase (#41; 1.6×) are components of the S-adenosyl methionine (SAM) cycle, while the remaining protein, serine hydroxymethyltransferase (#53; 2.2×), catalyzes the transfer of carbon into the SAM cycle, via folate. Because the cumulative function of these enzymes is to provide activated methyl groups for transfer to acceptors, the identity of the major methyl transferases and their substrates in fibres is an obvious question. In plants, potential acceptors of activated methyl groups include a wide variety of molecules, among them components of pectin or lignin [22]. Because the amount of lignin present in flax fibres is low in comparison to other types of schlerenchyma, particularly at the early stage of cell wall development associated with the snap point, [23,24], it seems unlikely that lignin is the major sink for methyl flux through the SAM cycle. Thus, pectin or other actively accumulating substances may be targets for SAM-mediated methylation in developing fibres.

### Membrane transport

Only a few proteins related to transport across membranes were detected in our study. This may be due in part to the difficulty of extracting and resolving certain membrane-associated proteins. Nevertheless, we identified a K^+ ^channel β-subunit was highly enriched (#97; 8.6×) in fibres, as well as two porins (#102, #102; 1.7×, 3.9×, respectively). The biological significance of the porins is unclear, however, increased expression of K^+ ^channels has been previously correlated with sucrose uptake in developing cotton fibres. Thus the strong enrichment of K^+ ^channel proteins we observed may reflect a similar process of the uptake of reduced carbon in flax fibres [25,26].

### Cytoskeleton and secretion

Structural components of the cytoskeleton, as well as proteins related to vesicle traffic, were also relatively more abundant in fibre protein extracts as compared to surrounding tissues. We observed relative enrichment of at least 1.5-fold of actin (#69, #70) and tubulin (#37) in fibres. These proteins may be enriched in fibres, as compared to cells of the non-fibre fraction, due in part to the differences in architecture and surface/volume ratios of these cells. Additionally, increased relative abundance of cytoskeleton proteins in fibres undergoing cell wall thickening may reflect the role of the cytoskeleton in deposition of cellulose and other cell wall components. An active secretory system, which delivers non-cellulosic polysaccharide components to the cell wall, is also expected to be present in developing flax fibres; the enrichment of myosin (#5, 2.5×; #6, 3.6×), dynamin-like proteins (#22, 3.1×), and GDP-dissociation inhibitor (#55, 2.0×; #56, 1.9×) in these cells is therefore consistent with developmental processes presumed to be active in the cells we sampled. We also note that other components of the cytoskeleton mentioned in a structural context above (i.e. actin and tubulin) may have additional functions specifically related to secretion and other aspects of secondary wall deposition [27-29].

### Protein and amino acid metabolism

Enzymes related to protein metabolism (e.g. protein synthesis and folding) were moderately enriched (1.5× – 2.7×) in fibres as compared to the non-fibre fraction. Two translation initiation factors were more abundant in the fibre sample: eIF-4A (#62, 1.6×) and eIF-5A (#114, 2.0×). Proteins in the eIF-4A family form part of the ribosomal machinery and are involved in binding and unwinding mRNA for translation, while some eIF-5A isoform family members have more diverse functions in cell division and related processes [30]. A translational elongation factor EF2 (#4, 2.5×) was also more abundant in fibres, while spots containing EF1á were similarly abundant (#67, 1.2×) or 2.3× fold less abundant (#54) in fibres as compared to the non-fibre fraction.

Heat shock proteins HSP60 (#29, 2.7×; #30, 1.5×; #32, 2.1×), HSP70 (#20, 1.9×; #21, 1.7×; #23, 2.0×), and HSP90 (#11, 1.7×) were also enriched in fibres. These proteins may function in the processes of cytosolic protein folding and protein import into mitochondria and chloroplasts, which are commonly associated with members of the HSP60, HSP70, and HSP90 families [31]. Additionally, because HSP70s have been shown to have specific functions in cell wall development in yeast, we cannot exclude the possibility that some of these proteins are active at the plasma membrane during the deposition of the flax fibre secondary wall [32,33].

### Miscellaneous

Several of the proteins we identified could not be classified into any of the larger functional categories we have already described. Eight of these proteins were enriched by 1.5× (p ≤ 0.05) or more in fibres, and may accordingly have specific roles in fibre development. These included annexins (#97, 2.2×; #98, 4.1×), enoyl-ACP reductase (#100, 2.1×), maturase K (#112, 3.4×), a 14-3-3 protein (#108, 2.6×), peroxidase (#85, 2.4×), and a protein kinase C inhibitor (#107, 2.8×). Among these, the enrichment of annexin in developing fibres is particularly interesting, given its previous association with cellulose synthase in structural and proteomic studies of cotton fibres [16,34].

### Comparison to transcriptomic analysis

The experimental approach used in the present study differs in many ways from our previously reported microarray analysis of flax stems [8]. Importantly, in the previous report, we did not dissect fibres away from other stem tissues; rather we compared transcript abundance in stem segments containing fibres at different stages of development. Therefore, a global comparison of these datasets is not warranted. Notwithstanding these limitations, we noted that three carbohydrate-related enzymes were detected both as proteins enriched in fibres from the snap-point region of the stem, and previously as transcripts expressed in the region of the stem containing the snap-point, including β-galactosidase (#12–16, #64), fructokinase (#93, #94), and GME (#101) (Table 1). In the transcriptomic data, β-galactosidase and fructokinase were significantly more abundant in the region of the snap-point as compared to segments from nearer either the apex or base of the stem, while GME showed highest transcript abundance in the apical-most segment, which may be due to differences in the turnover of these various gene products. On the other hand, our previous work also identified many other snap-point enriched transcripts that were not detected as proteins in the previous study. These include arabinogalactan proteins and lipid transfer proteins that were further demonstrated by qRT-PCR to be enriched specifically in the phloem tissues of the snap-point, as compared to leaves or the xylem of stems. Discrepancies between transcriptomic and proteomic analyses have been previously documented by ourselves and others, and are presumably due to differences in efficiencies of extraction and detection of various proteins, among many other technical and biological factors [35]. For example, Bayer et al. specifically noted under representation of AGPs and other cell wall proteins within their proteomic analysis, due possibly to the high degree of glycosylation of these proteins [12]. Thus, it appears likely that a comprehensive description of gene expression within developing flax fibres cannot be provided by either transcript or protein profiling, alone, but instead the results of many different experimental approaches must be considered together.

## Conclusion

We have described a differential proteomic profile of a single plant cell type at a well-defined developmental stage, during which secondary cell wall biogenesis is occurring. The fibre-enriched proteins we identified are consistent with the dynamic process of secondary wall deposition previously suggested by histological and biochemical analyses, and particularly the importance of galactans and the secretory pathway in this process [6]. The apparent abundance of amylase suggests that starch may be an unappreciated source of materials for cell wall biogenesis. Furthermore, our observations confirm previous reports that correlate accumulation of proteins such as annexins, and specific heat shock proteins with secondary cell wall deposition [6,16,33]. Together, the proteins we have identified in this study provide a basis for better understanding the unique properties of phloem fibre secondary cell walls, and define targets for detailed genetic and biochemical analyses in future.

## Methods

### Plant material

Fibres (i.e. individual cells) and surrounding, non-fibre cells of the cortex were isolated from the stems of *Linum usitatissimum *L., var. Norlin. A total of 495 plants were harvested from four independently grown populations. Seeds were sown two per 10 cm pot and grown as previously described [8]. After 3 weeks of growth, the mean distance from the apex to snap-point was 5.9 cm, with mean plant height of 19 cm. A 3 cm segment of stem, spanning from 2 cm to 5 cm below the snap-point, was further dissected to separate the individual fibres and surrounding non-fibre cells of the cortex (i.e. "the non-fibre fraction", consisting predominantly of parenchyma, sieve elements, and companion cells, but excluding epidermis, xylem and pith) for proteomic analysis. After dissection, fibres and surrounding tissues were rinsed in deionized water, blotted, then frozen in liquid nitrogen, and stored at -80°C.

### Protein isolation from tissues

Tissues were ground to a powder in liquid nitrogen and then further ground for one minute in 1 mL cold TCA/acetone buffer (20 mM DTT, 10% trichloroacetic acid in cold acetone). Homogenates were transferred with an additional 1 mL of buffer to microcentrifuge tubes and were allowed to precipitate overnight at -20°C. After centrifugation (13000 rpm, 10°C, 15 minutes), pellets were rinsed once with 1 mL 20 mM DTT in acetone for 1 h at -20°C, then pellets were left to dry at -20°C for 2 h, and dissolved in 200 μL of urea/thiourea buffer (7 M urea, 2 M thiourea, 4% (w/v) CHAPS, 30 mM Tris-Cl) by vortexing at room temperature for 30 minutes. The solution was clarified by centrifugation (13000 rpm, 17°C, 5 minutes) and supernatants were further processed by using the 2D Clean-Up Kit (Amersham Biosciences). Precipitates were re-dissolved in 60 μl of the urea/thiourea buffer, and concentrations of the protein samples were determined using the 2D Quant Kit (Amersham Biosciences) and NanoDrop^® ^ND-1000 spectrophotometer (NanoDrop Technologies) against a BSA standard curve.

### Fluorescent labeling of proteins

Four independent pools of approximately 125 plants each were grown in nominally identical conditions that were spatially and temporally separated from each other. Proteins were isolated separately from tissues dissected from each pool of plants, to produce four paired protein samples from fibres and the non-fibre fraction, where each pair of samples was biologically independent from every other pair. We labeled each 30 μg protein sample (pH adjusted to 8.5) with 240 pmol of Cy2, Cy3 or Cy5 fluorescent dyes, using the CyDye™ DiGE fluors (minimal dyes) labeling kit (Amersham Biosciences). Labeling reactions were stopped by the addition of 1 μl of 10 mM lysine to each tube, and after a further 10-minute incubation on ice, the volume of each sample was doubled with the addition of a sample buffer (7 M urea, 2 M thiourea, 2% (v/v) ampholyte, 2% (w/v) DTT, 4% (w/v) CHAPS) to ready the samples for IEF. Labeled samples were mixed together as stated in Table 2 to create four analytical gels, with each gel containing an internal standard and both tissue samples. The internal standard is prepared by mixing equal masses of protein extracts from fibre and non-fibre fractions of each biologically independent harvest.

**Table 2:** Experimental design relative to labeling and sample loading of analytical gels.

gel	Cy2 labeled	Cy3 labeled	Cy5 labeled
1	internal standard #1 30 μg	fibre sample #1 30 μg	non-fibre sample #1 30 μg
2	internal standard #2 30 μg	fibre sample #2 30 μg	non-fibre sample #2 30 μg
3	internal standard #3 30 μg	non-fibre sample #3 30 μg	fibre sample #3 30 μg
4	internal standard #4 30 μg	non-fibre sample #4 30 μg	fibre sample #4 30 μg

Note: each gel contains proteins from a unique pool (#1–#4) of independently grown plants. The Cy2-labeled internal standard is a mixture of equal masses of proteins from fibre and non-fibre samples.

### 2DE of CyDye labeled protein mixtures

All subsequent handling and separation steps for 2DE were conducted away from light. 24 cm, 3–10 NL Immobiline™ drystrips (Amersham Biosciences) were passively re-hydated for 10 h in (8 M urea, 4% (w/v) CHAPS, 1% (v/v) ampholytes 3–10, 13 mM DTT, trace bromophenol blue). A total of 56 kVh at 20°C was used to focus the proteins using an IPGphor™ II (Amersham Biosciences). Paper wicks on the basic end were spiked with 13 mM DTT and were changed three times during the run. Following IEF, strips were equilibrated for SDS-PAGE separation by gentle agitation for 15 minutes in 6 M urea, 50 mM tris-Cl (pH 8.8), 30% (v/v) glycerol, 2% (w/v) SDS, trace bromophenol blue plus 0.5% (w/v) DTT, followed by 15 minutes in the same solution with 4.5% (w/v) IAA instead of DTT. After equilibration, the strips were sealed onto the top edge of self-cast, large-format, 12.5% acrylamide gels using sealing solution (1% low-melt agarose, trace bromophenol blue in 1X running buffer). The four analytical gels were separated by molecular weight during SDS-PAGE, simultaneously, using the Ettan™ Dalt *six *(Amersham Biosciences). The gels were run at 2 W/gel for 30 minutes then 8 W/gel until the bromophenol blue dye front just touched the end of the gels.

### Imaging and analysis

Fluorescently labeled gels were imaged at 100 μm resolution with PMT voltage between 50000 and 63558 V. DeCyder™ 6.5 (Amersham Biosciences) was used to match, normalize, and statistically analyze spots. After in-gel normalization using Differential In-gel Analysis (DIA), the Biological Variation Analysis (BVA) module was used for statistical analysis and normalization across all analytical gels.

### Spot-picking and tryptic digestion of proteins

Preparative gels, loaded with about 125 μg of protein, were post-stained with Deep Purple™ total protein stain (Amersham Biosciences) and spot-matched to the analytical gels. Gel spot excision and subsequent tryptic digestion were conducted using an Ettan™ Spot-picker (Amersham Biosciences) and ProteomeWorks™ MassPREP™ robotic handling station (Bio-Rad Laboratories and Waters corporations), resulting in peptides in a final extraction solution of 2% ACN, 0.1% formic acid in H_2_0.

### Protein identification

LC MS/MS analysis was performed using an online 1100 series XCT Ion trap (Agilent Technologies). The autosampler injected 18 μL of each sample onto an enrichment column (Zorbax 300SB-C18 5 μm 5 × 0.3 mm) that connected to a second column (Zorbax 300SB-C18 5 μm 150 × 0.3 mm) in a peptide-separation gradient that started at 85% solvent A (0.1% formic acid in H_2_O) and ended at 55% solvent B (0.1% formic acid, 5% H_2_O in ACN) over a 42 minute span. This was followed by 10 minutes of 90% solvent B to cleanse the columns before returning to 97% solvent A for the next sample. The MS ran a 300–2200 m/z scan followed by MS/MS analysis of the most intense ions. Raw spectral data was processed into Mascot Generic File (.mgf) format using the default method in the ChemStation Data Analysis module and ion searches were completed in MASCOT [36] with the search parameters of: peptide tolerance of 2 Da, parent ion tolerance of 0.8 m/z, ion charge of +1, +2 and +3.

## List of abbreviations

1C: one-carbon; DiGE: differential gel electrophoresis; dTDP: thymine diphosphate deoxynucleotide; EF: translational elongation factor; eIF: translational initiation factor; GDP: guanine diphosphate; SAM: S-adenosyl methionine; UDP: uridine diphosphate.

## Authors' contributions

NSCH designed and conducted all experiments, including operation of the mass spectrometer and interpretation of mass spectra, and wrote the original draft of this manuscript. MKD supervised all research, and contributed to writing and editing of the manuscript.

## Supplementary Material

Additional file 1 Additional information (e.g. peptide sequences; M_r_, pI of database matches, PFAM domains) on spot identifications that was not otherwise conveyed in Table 1. Click here for file
